# Shunt: pathophysiological mechanisms and clinical implications of ineffective circulating volume

**DOI:** 10.3389/fphys.2026.1939745

**Published:** 2026-09-10

**Authors:** Shenghui Miao, Jiatao Yuan, Zhaokun Fan, Zhiqiang Gu, Zhirong Zhang

**Affiliations:** 1 Department of Intensive Care Unit, The First Affiliated Hospital of Zhejiang Chinese Medical University (Zhejiang Provincial Hospital of Chinese Medicine), Hangzhou, Zhejiang, China; 2 Department of Data Science, Brown University, Providence, RI, United States; 3 The Third People’s Hospital of Hangzhou Lin’an District, Hangzhou, Zhejiang, China

**Keywords:** distributive shock, ineffective circulating volume, microcirculation, pulmonary shunt, systemic shunt

## Abstract

The classical concept of shunt confines the phenomenon to the pulmonary circulation, leaving microcirculatory flow maldistribution in distributive shock outside existing terminology. Tissue hypoxia can persist despite normal cardiac output; current hemodynamic stability criteria incorporate no microcirculatory dimension. This review addresses both gaps by proposing a unifying framework. We propose ineffective circulating volume (ICV) as blood flow that fails to achieve effective gas exchange, whether at the alveolar-blood interface (pulmonary shunt) or the blood-cell interface (systemic shunt). Pulmonary shunt and septic microcirculatory heterogeneity are established phenomena, whereas their unification as ICV—particularly the term systemic “functional shunt”—is a hypothesis requiring clinical validation. Anatomical shunt involves fixed structural bypasses correctable surgically; functional abnormalities reflect failure of active distribution mechanisms—hypoxic pulmonary vasoconstriction suppressed by inflammatory mediators, and conducted responses disrupted by endothelial injury. Supportive interventions may improve their physiological consequences, whereas durable reversal depends on resolution or treatment of the underlying disease. The ICV framework subdivides circulatory hypoxia into a macrocirculatory sublayer (global oxygen delivery insufficiency) and a microcirculatory sublayer (flow maldistribution), the latter potentially persisting even when macrocirculatory parameters are normalised. Acute respiratory distress syndrome and distributive shock may share three upstream molecular mechanisms—glycocalyx degradation, spatially heterogeneous inducible nitric oxide synthase overexpression, and neutrophil extracellular traps—that could impair regulatory systems at both interfaces. Changes in sepsis resuscitation targets are compatible with, but do not validate, the ICV framework’s emphasis on macro-microcirculatory uncoupling. No study has simultaneously quantified shunt at both interfaces in the same patient cohort, and no randomised trial targeting the shared upstream mechanisms has used dual-interface ICV as a combined primary endpoint. Translating the ICV framework into prospectively defined clinical endpoints remains an open methodological challenge.

## 1 Introduction: from venous admixture to ineffective circulating volume

In classical physiology, “shunt” refers to venous blood bypassing functional alveolar-capillary units, entering the left heart without oxygenation and causing hypoxemia—quantified by the shunt fraction (*Q_s_/Q_t_*) via the Riley-Cournand model (Radermacher et al., 2017; Albert and Jobe, 2012). This framework has two limitations. First, “shunt” is almost exclusively assigned to the pulmonary circulation, although non-nutritive microcirculatory flow can likewise be ineffective (Elbers and Ince, 2006; Ince and Sinaasappel, 1999). Second, tissue hypoxia may persist when global oxygen delivery is normal or elevated: in distributive shock, cardiac output is often supranormal and mixed venous oxygen saturation (SvO_2_) may paradoxically increase (Pries et al., 2010; Ince and Sinaasappel, 1999). We use “systemic shunt” as a functional analogy for this maldistribution, not as accepted nomenclature or a measured shunt fraction equivalent to *Q_s_/Q_t_*.

We propose reinterpreting “shunt” through the concept of ineffective circulating volume (ICV)—blood that fails to achieve effective gas exchange, whether at the alveolar-blood interface (pulmonary shunt) or the blood-cell interface (systemic shunt). Cardiac output and effective gas exchange capacity are therefore not interchangeable. ICV is a conceptual framework rather than a directly measured intravascular compartment. It further divides by structural basis into anatomical shunt (fixed abnormalities, potentially correctable by anatomy-specific intervention) and functional shunt (physiological dysregulation whose magnitude or consequences may be modified supportively, while durable reversal generally requires treatment of the underlying disease) (**Table 1**).

**Table 1:** Comparison of pulmonary shunt and systemic shunt.

Category	Pulmonary shunt	Systemic shunt
Interface	Alveolar-blood gas exchange	Blood-cell gas exchange
Mechanism	Anatomical or functional (V/Q=0)	Microcirculatory functional short-circuit
Main consequence	Refractory arterial hypoxemia	Circulatory hypoxia
Typical disease	ARDS*^a^*, pulmonary AVM*^b^*	Sepsis, distributive shock
Key indices	SaO_2_↓, PaO_2_/FiO_2_↓	Low or inappropriately normal O_2_ER may suggest extraction impairment; high O_2_ER suggests global DO_2_ insufficiency

*^a^*ARDS, acute respiratory distress syndrome; *^b^*AVM, arteriovenous malformation.

## 2 Pulmonary shunt: pathophysiology, monitoring, and treatment

### 2.1 Mechanisms and pathophysiology

Pulmonary shunt reduces arterial oxygen content when venous blood enters the left heart without completing O_2_–CO_2_ exchange. Anatomical shunt results from fixed structural bypasses—patent foramen ovale (PFO), pulmonary arteriovenous malformations (PAVM), and intrapulmonary anastomoses—whose quantitative contributions vary (Homma et al., 2016; Velthuis et al., 2015). Functional shunt is more prevalent in critical care: consolidation, atelectasis, or exudate eliminate alveolar ventilation while capillary perfusion is maintained, forming V/Q=0 zones that are physiologically equivalent to anatomical shunt (Albert and Jobe, 2012; Gierhardt et al., 2021). The hallmark is refractory hypoxemia unresponsive to increased FiO_2_.

**Figure 1:**
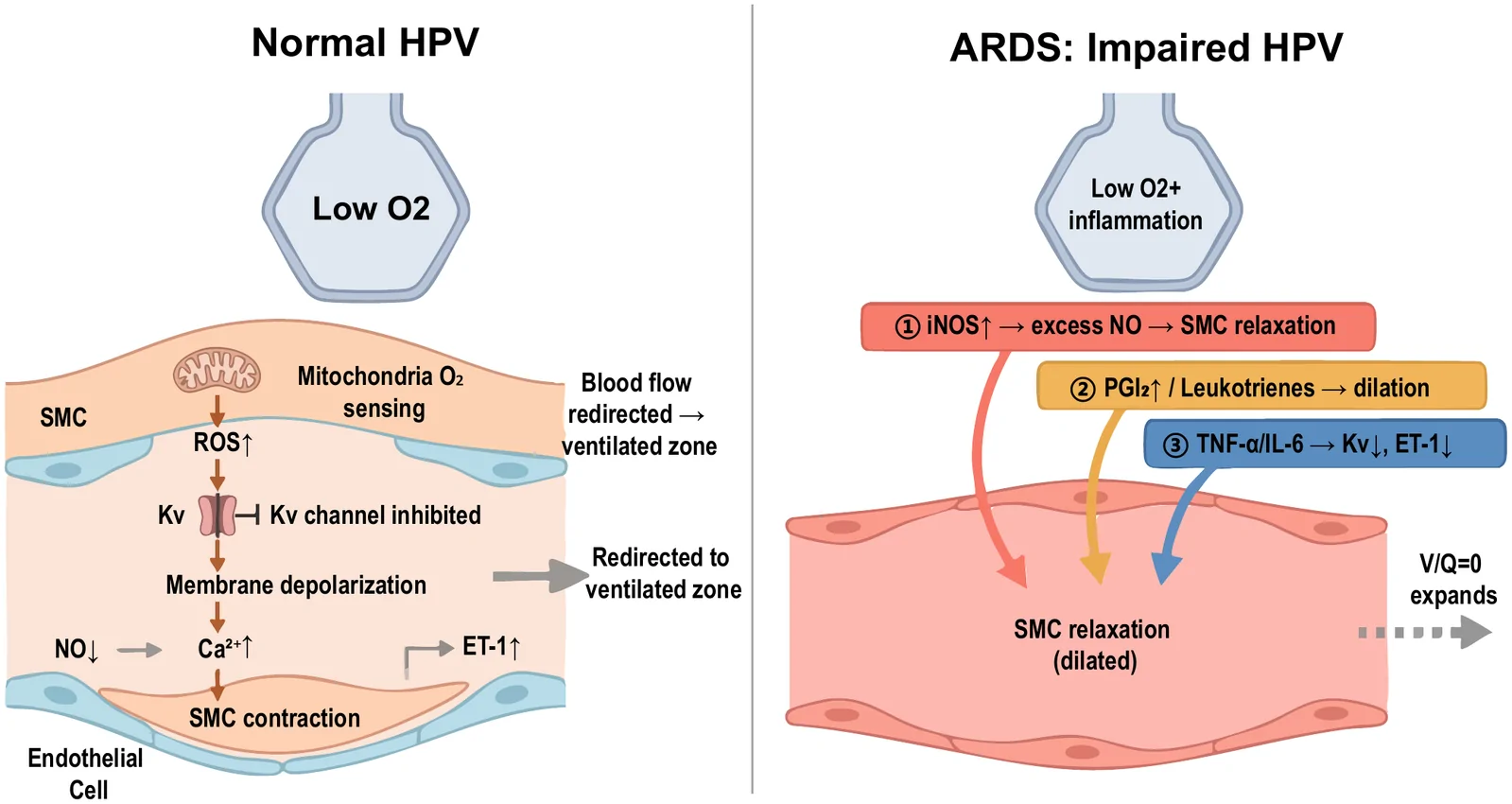
Normal HPV mechanism and impaired HPV in ARDS. Left: Under normoxia, mitochondrial O_2_ sensing maintains K_v_ channel activity in pulmonary arterial SMCs. Alveolar hypoxia inhibits K_v_, triggering depolarization, Ca^2+^ influx, SMC contraction, and blood flow redirection toward ventilated zones (HPV). Endothelial eNOS-derived NO and ET-1 modulate vasomotor tone. Right: In ARDS, three mechanisms may impair HPV: (1) iNOS-derived excess NO causes SMC over-relaxation via guanylate cyclase-cGMP; (2) elevated PGI_2_ and leukotrienes promote vasodilation; (3) TNF-*α*/IL-6 suppress K_v_ and reduce ET-1. The result may be persistent perfusion of non-ventilated alveoli, expanding V/Q=0 shunt. ARDS, acute respiratory distress syndrome; ET-1, endothelin-1; HPV, hypoxic pulmonary vasoconstriction; iNOS, inducible nitric oxide synthase; K_v_, voltage-gated potassium channel; NO, nitric oxide; PGI_2_, prostacyclin; ROS, reactive oxygen species; SMC, smooth muscle cell; V/Q, ventilation-perfusion ratio.

In normal lungs, hypoxic pulmonary vasoconstriction (HPV) is the key endogenous defence against functional shunt (**Figure 1**): alveolar hypoxia triggers mitochondrial O_2_ sensing, ROS-mediated K_v_ channel inhibition, membrane depolarization, Ca^2+^ influx, and smooth muscle cell (SMC) contraction, redirecting blood toward ventilated zones (Gierhardt et al., 2021). In ARDS, three convergent mechanisms may impair HPV: (1) iNOS-derived excess NO persistently activates guanylate cyclase-cGMP, causing SMC over-relaxation; (2) PGI_2_ excess and leukotriene-mediated dilation oppose HPV; (3) TNF-*α* and IL-6 inhibit K_v_ channel function and impair endothelin-1 (ET-1) secretion (Gierhardt et al., 2021). The net effect may be persistent perfusion of non-ventilated alveoli with expansion of V/Q=0 shunt. Pulmonary hypertension in ARDS can additionally recruit PFO right-to-left shunting; transpulmonary bubble transit (TPBT) is detected in approximately 20-26% of cases (Boissier et al., 2015), underscoring that anatomical and functional shunt coexist.

### 2.2 Monitoring of pulmonary shunt

**Figure 2:**
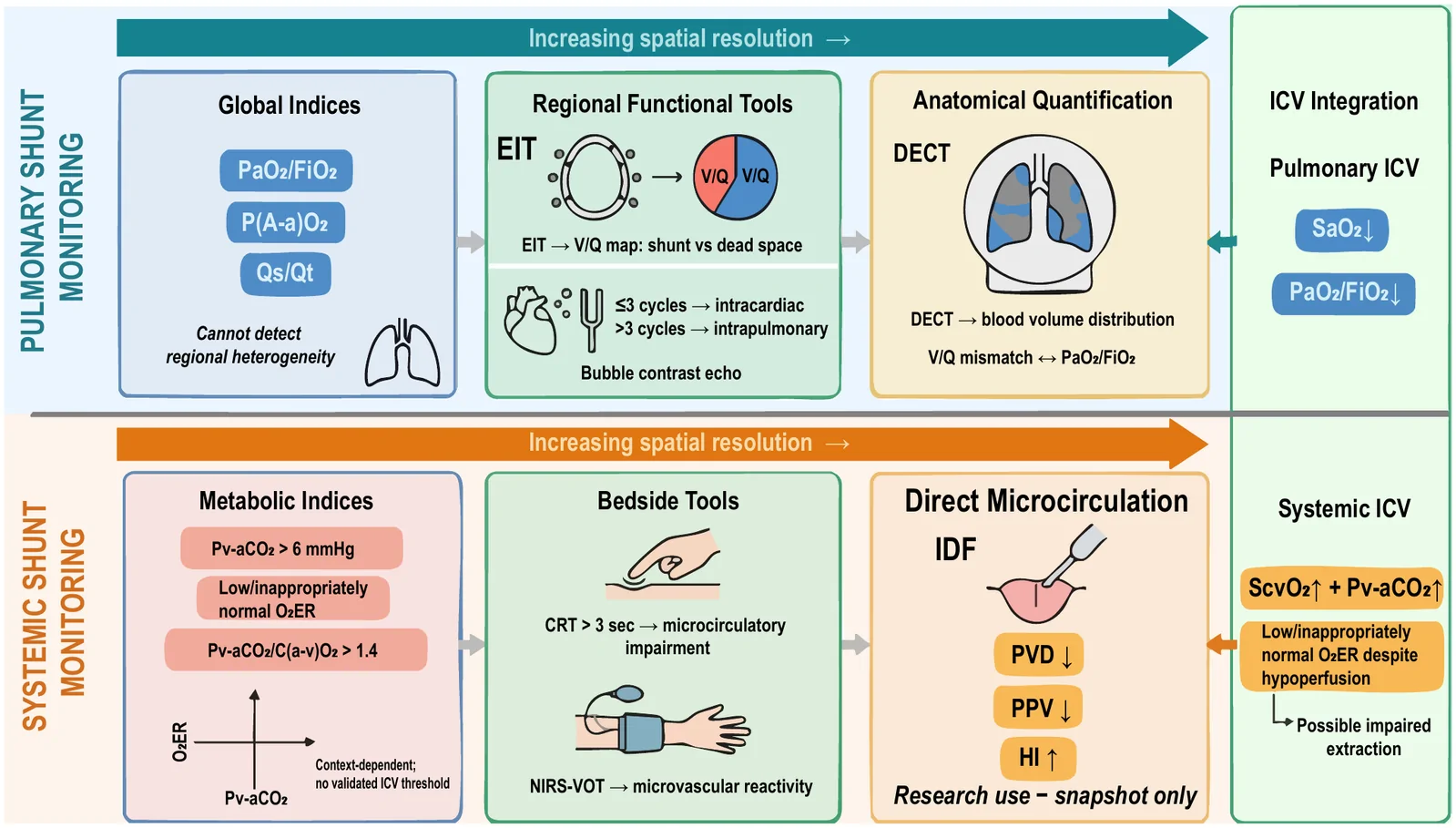
Hierarchical monitoring framework for pulmonary and systemic shunt within the ICV paradigm. Upper half—Pulmonary shunt: Global indices (PaO_2_/FiO_2_, P(A-a)O_2_, Q_s_/Q_t_) lack regional resolution. EIT generates real-time V/Q maps. Bubble contrast echocardiography discriminates intracardiac from intrapulmonary shunting. DECT quantifies regional gas-blood mismatch. *Lower half—Systemic shunt*: Low or inappropriately normal O_2_ER with elevated P_v-a_CO_2_ may support impaired extraction within the proposed ICV phenotype; high O_2_ER instead suggests global DO_2_ insufficiency. These indices are non-specific. IDF microscopy quantifies PVD, PPV, and HI, while CRT and NIRS-VOT provide adjunctive bedside assessment. Arrow denotes increasing spatial resolution. C(a-v)O_2_, arteriovenous oxygen content difference; CRT, capillary refill time; DECT, dual-energy computed tomography; EIT, electrical impedance tomography; HI, heterogeneity index; ICV, ineffective circulating volume; IDF, incident dark-field microscopy; NIRS-VOT, near-infrared spectroscopy with vascular occlusion test; O_2_ER, oxygen extraction ratio; P(A-a)O_2_, alveolar-arterial oxygen gradient; PPV, proportion of perfused vessels; PVD, perfused vessel density; Q_s_/Q_t_, shunt fraction; ScvO_2_, central venous oxygen saturation; P_v-a_CO_2_, venoarterial CO_2_ difference.

Traditional blood gas indices (PaO_2_/FiO_2_, P(A-a)O_2_, *Q_s_/Q_t_*) reflect global gas exchange averages and are confounded by cardiac output, haemoglobin, and FiO_2_ (Feiner and Weiskopf, 2017). Electrical impedance tomography (EIT) generates bedside regional ventilation maps and, with specialised protocols, estimates perfusion; pre-prone dependent-zone shunt fraction *<* 8.4% independently predicts prone responsiveness (sensitivity 94%), and V/Q matching improvement during proning is independently associated with reduced ICU mortality (Leali et al., 2024; Wu et al., 2025; Wang et al., 2025). Bubble contrast echocardiography discriminates intracardiac (≤3 cardiac cycles) from intrapulmonary (*>*3 cycles) shunting (Boissier et al., 2015), and dual-energy CT (DECT) quantifies gas-blood volume mismatch, which correlates inversely with PaO_2_/FiO_2_ (Busana et al., 2024). EIT is non-invasive and repeatable but requires dedicated equipment, trained acquisition, and attention to motion and belt-position artefacts; DECT provides greater spatial detail but requires transport, radiation, and contrast exposure (Frerichs et al., 2017). The hierarchical structure of these tools within the ICV framework is shown in **Figure 2**.

### 2.3 Treatment of pulmonary shunt

Prone positioning promotes dorsal recruitment and optimises V/Q distribution; early application (≤7 days) reduces mismatch, whereas late application may paradoxically increase shunt (Wang et al., 2022; Yuan et al., 2023). PEEP prevents end-expiratory collapse; its net effect on shunt depends on recruitability— overdistension/recruitment (O/R) ratio <15% typically improves V/Q matching (Karbing et al., 2020). For refractory hypoxaemia, VV-ECMO enables ultra-protective ventilation; prone-ECMO combinations further reduce intrapulmonary shunt (Quintel et al., 2020). Inhaled vasodilators (iNO, prostaglandins) selectively dilate vessels in ventilated zones; almitrine reinforces HPV and improved oxygenation in ∼66% of COVID-19 ARDS patients, though at the cost of right ventricular strain at higher doses (Kaisers et al., 2003; Caplan et al., 2021). Sildenafil worsens shunt fraction and is contraindicated in ARDS (Cornet et al., 2010). Anatomical shunts are managed interventionally (PFO closure, antibiotic prophylaxis for PAVM) or via liver transplantation for hepatopulmonary syndrome (Homma et al., 2016). These interventions may reduce the shunt fraction or its physiological consequences without necessarily reversing the underlying inflammatory lung injury.

Pulmonary shunt depresses systemic DO_2_ through reduced arterial oxygen content; compensatory cardiac output increases paradoxically amplify shunt-zone blood volume, further diluting CaO_2_. The two interfaces are therefore not hemodynamically independent.

## 3 Systemic shunt: pathophysiology, monitoring, and treatment

**Figure 3:**
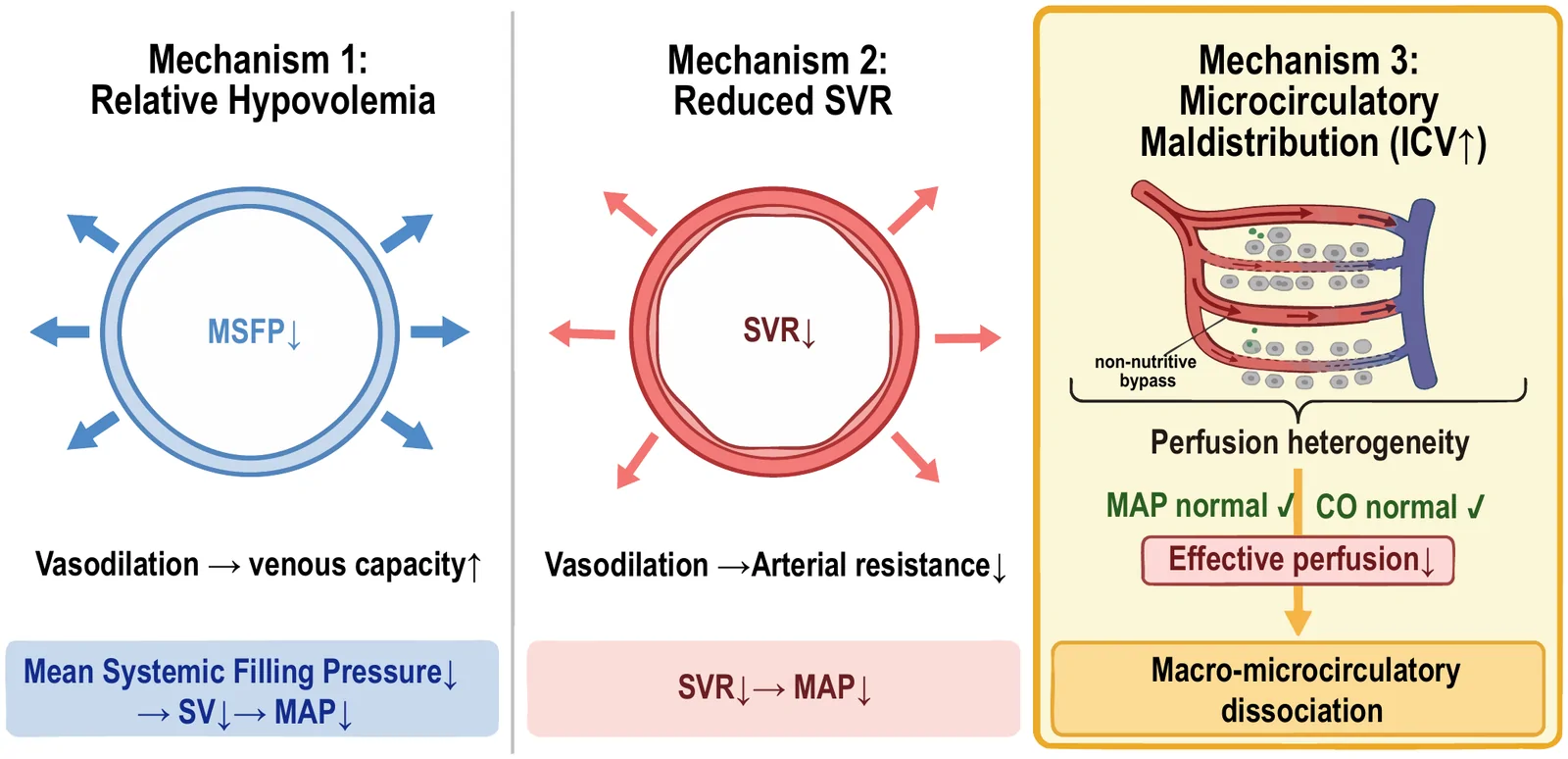
Three pathophysiological mechanisms of distributive shock within the ICV framework. Mechanism 1—Relative hypovolemia: Systemic vasodilation increases venous capacitance, reducing MSFP, stroke volume, and MAP. Mechanism 2—Reduced SVR: Arteriolar vasodilation decreases SVR and MAP without necessarily reducing cardiac output. Mechanism 3—Microcirculatory maldistribution (proposed ICV interpretation): Even when MAP and cardiac output are normalised, perfusion heterogeneity may contribute to loss of hemodynamic coherence and reduce effective tissue perfusion despite adequate global flow. ICV, ineffective circulating volume; MAP, mean arterial pressure; MSFP, mean systemic filling pressure; SVR, systemic vascular resistance.

The ICV framework adds a third mechanism to the conventional two macrocirculatory explanations of distributive shock (vasodilation-driven relative hypovolaemia; reduced SVR): microcirculatory flow maldistribution, which may cause tissue hypoxia even when macrocirculatory parameters are normalised (**Figure 3**) (Elbers and Ince, 2006; Ince, 2005; Vincent and De Backer, 2013). Ince’s concept of “hemodynamic coherence” captures this loss of correspondence between macro- and microcirculatory responses (Ince, 2015). Loss of hemodynamic coherence may help explain why normalising global targets did not improve outcomes in ProCESS, ARISE, and ProMISe, although these trials did not directly test the ICV hypothesis (Yealy et al., 2014; Peake et al., 2014; Mouncey et al., 2015).

### 3.1 Mechanisms of microcirculatory functional shunt

**Figure 4:**
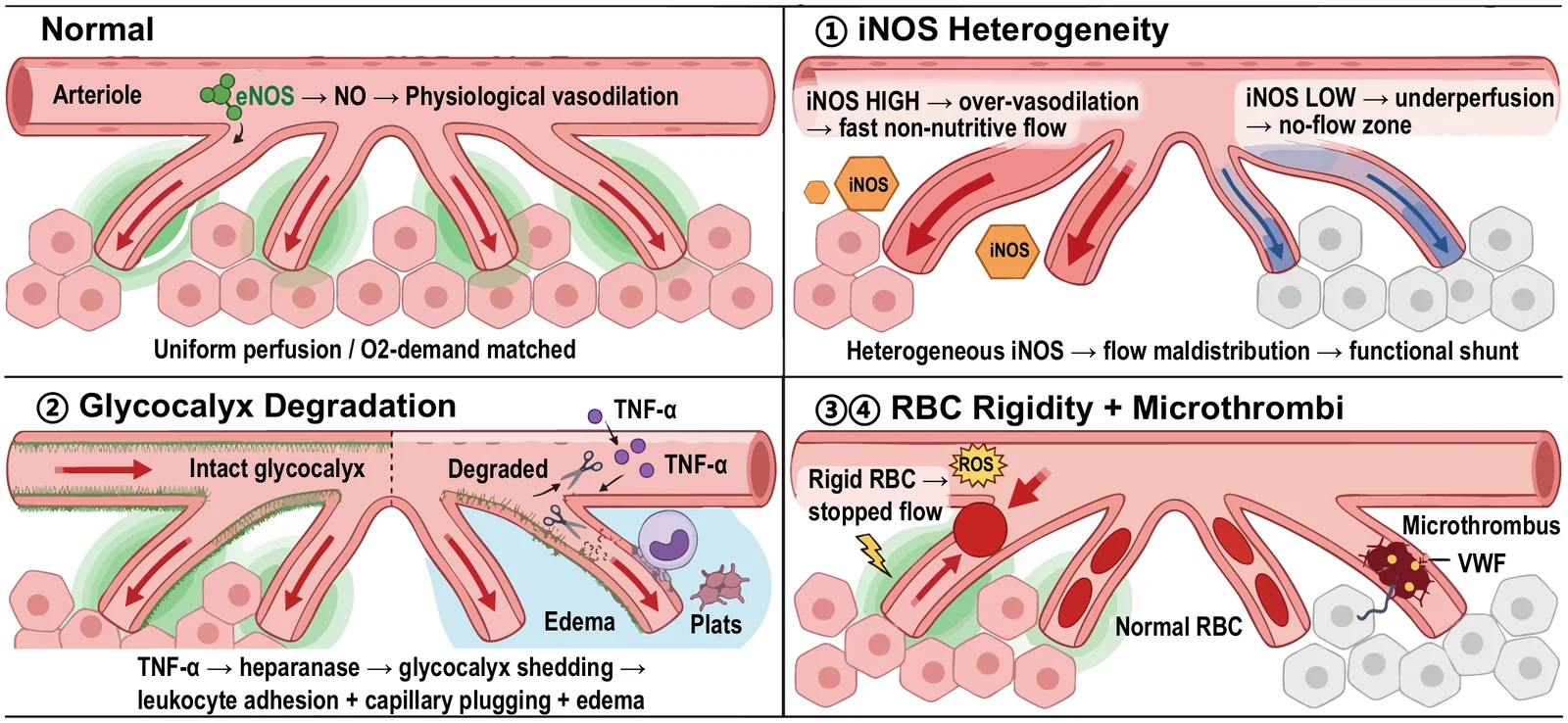
Four molecular mechanisms of microcirculatory maldistribution in septic shock. Normal (top left): eNOS-derived NO maintains uniform capillary perfusion matched to O_2_ demand. *iNOS heterogeneity* (top right): Spatially heterogeneous iNOS overexpression drives over-vasodilation in highexpression zones (fast non-nutritive flow) and underperfusion in low-expression zones. *Glycocalyx degradation* (bottom left): TNF-*α* activates heparanase; leukocyte adhesion, plugging, and oedema impair nutritive perfusion. *RBC rigidity and microthrombi* (bottom right): Oxidative stress reduces erythrocyte deformability; VWF-platelet microthrombus formation occludes capillary segments. Together these mechanisms can reduce PVD, increase HI, and contribute to loss of hemodynamic coherence; their interpretation as systemic ICV remains hypothetical. eNOS, endothelial nitric oxide synthase; HI, heterogeneity index; iNOS, inducible nitric oxide synthase; NO, nitric oxide; PVD, perfused vessel density; RBC, red blood cell; TNF-*α*, tumour necrosis factor-alpha; VWF, von Willebrand factor.

In normal microcirculation, metabolic signals (local hypoxia, adenosine, CO_2_) are retrogradely transmitted via conducted responses to upstream arterioles, driving targeted dilation that recruits blood to high-demand zones (Pries et al., 2010; Segal, 2005). Red blood cells additionally act as PO_2_ sensors: haemoglobin conformation changes release nitrosothiol and ATP, inducing microvascular dilation (Bateman et al., 2003). In septic shock, this regulatory capacity is disrupted through four superimposed mechanisms (**Figure 4**): (1) spatially heterogeneous iNOS overexpression drives over-vasodilation in high-expression beds (fast non-nutritive flow) while low-expression areas are underperfused (no-flow zones); (2) glycocalyx degradation by TNF-*α*-activated heparanase causes syndecan-1 shedding, leukocyte adhesion, and capillary permeability increase; (3) oxidative stress reduces RBC deformability, causing stopped flow at 3-5*µ*m capillary entrances; (4) glycocalyx loss exposes tissue factor, initiating platelet-mediated microthrombus formation (Bateman et al., 2003; Joffre et al., 2020; Uchimido et al., 2019). These mechanisms can reduce perfused vessel density (PVD), increase perfusion heterogeneity, and contribute to loss of hemodynamic coherence.

### 3.2 Monitoring and assessment of systemic shunt

Systemic shunt monitoring combines indirect metabolic indices with direct microcirculation imaging (**Figure 2**). O_2_ER and C(a-v)O_2_ are the primary Fick-principle indices; SvO_2_/ScvO_2_ is a composite that does not directly reflect extraction efficiency (Vallet et al., 2013). In distributive shock, low or inappropriately normal O_2_ER despite evidence of hypoperfusion may reflect impaired extraction from flow maldistribution or cytopathic hypoxia, whereas high O_2_ER more typically indicates global DO_2_ insufficiency. Neither pattern is specific for ICV, and no validated O_2_ER threshold diagnoses systemic shunt. P_v-a_CO_2_ (normal *<* 6mmHg) reflects whether blood flow is adequate relative to CO_2_ production; P_v-a_CO_2_/C(a-v)O_2_ *>*1.4mmHg·dl/ml has been associated with anaerobic metabolism (Mesquida et al., 2015). Thus, low or inappropriately normal O_2_ER with elevated P_v-a_CO_2_ may support, but cannot establish, the proposed systemic ICV phenotype.

Near-infrared spectroscopy with a vascular occlusion test (NIRS-VOT) measures peripheral tissue oxygen saturation (StO_2_); the post-occlusion reoxygenation slope provides an index of microvascular recruitment and reactivity. Abnormal responses have been reported in sepsis and critical illness (Lima et al., 2009; Donati et al., 2016). NIRS-VOT is non-invasive but samples superficial regional tissue and is affected by probe site, tissue thickness, oedema, vasoactive therapy, device, and protocol. It should therefore be considered an adjunct rather than a direct measure of systemic ICV.

Sublingual IDF/SDF microscopy directly quantifies PVD, proportion of perfused vessels (PPV), and heterogeneity index (HI); decreased PVD and elevated HI independently associate with organ dysfunction and mortality, persisting even after macrocirculatory normalisation (Sanfilippo et al., 2022; Aykut et al., 2015). Its routine use is limited by operator-dependent acquisition, sublingual sampling, artefacts, and the absence of validated treatment thresholds (Ince et al., 2018). Capillary refill time (CRT) *>* 3 seconds associates with sublingual microcirculatory abnormalities; ANDROMEDA-SHOCK evaluated CRT-guided resuscitation (Hernandez et al., 2019a), and the 2026 SSC guidelines suggest CRT as an adjunct to other measures of perfusion (Prescott et al., 2026). However, CRT may remain normal in hyperdynamic distributive shock despite substantial microcirculatory maldistribution (Kanoore Edul et al., 2016)—within the ICV framework, a normal CRT does not exclude elevated systemic ICV.

### 3.3 Treatment of systemic shunt

Tissue hypoxia from systemic shunt is spatially heterogeneous: bypassed zones accumulate lactate anaerobically while hyperperfused zones may fail to extract oxygen due to cytopathic hypoxia (Elbers and Ince, 2006). Persistently elevated lactate despite adequate DO_2_ may be consistent with this pattern, but adrenergic glycolysis and impaired clearance are alternative explanations. Three large-scale RCTs (ProCESS, ARISE, ProMISe; *n* = 4,183 combined) showed ScvO_2_-targeted EGDT conferred no benefit over standard care (Yealy et al., 2014; Peake et al., 2014; Mouncey et al., 2015). Microcirculatory maldistribution is one possible interpretation of the limited specificity of ScvO_2_, but these trials did not test the ICV framework. Current SSC guidance instead supports serial lactate, dynamic flow assessment, and CRT as an adjunct (Evans et al., 2021; Prescott et al., 2026).

When microcirculatory functional shunt is established, additional fluid may enter non-nutritive pathways and worsen tissue oedema (Ince, 2015; Hjortrup et al., 2016); vasopressors can restore perfusion pressure but may not correct spatial maldistribution (Hernandez et al., 2019b). Dobutamine may improve sublingual capillary perfusion independently of cardiac index changes, though responses are heterogeneous (De Backer et al., 2006). Trials of microcirculatory vasodilators (nitroglycerin, inhaled NO) have not established outcome benefit (Spronk et al., 2002; Boerma et al., 2010). Supportive treatment may improve pressure, flow, or the consequences of maldistribution, whereas source control and treatment of the underlying inflammatory process address its upstream drivers. Whether P_v-a_CO_2_/C(a-v)O_2_, CRT, and PVD can be combined into a composite resuscitation endpoint remains untested.

## 4 Shared mechanisms and functional analogy at both interfaces

ARDS and distributive shock share a functional analogy: ARDS generates V/Q=0 mismatch (ventilation absent, perfusion preserved), whereas distributive shock can generate perfusion-without-extraction mismatch (flow present, metabolic coupling impaired). The systemic “pO_2_ gap” between intestinal microvascular and venous oxygen partial pressures observed in endotoxaemia is analogous to, but not quantitatively equivalent to, the pulmonary P(A-a)O_2_ gap (Ince and Sinaasappel, 1999). When both coexist in sepsis-associated ARDS, abnormalities at the two interfaces may compound impaired oxygen transfer and utilization.

### 4.1 HPV and conducted response as parallel regulatory mechanisms

HPV and the systemic microvascular conducted response provide a functional regulatory analogy. HPV uses alveolar hypoxia to withdraw blood from ineffectively ventilated zones, whereas the conducted response uses tissue hypoxia, adenosine, and CO_2_ to recruit blood to high-metabolic-demand zones (Pries et al., 2010; Segal, 2005). In sepsis, iNOS overexpression and glycocalyx degradation may impair both processes. Supportive interventions can modify flow distribution or its consequences, while durable recovery of the regulatory lesion depends on the course and treatment of the primary inflammatory injury.

### 4.2 Shared molecular mechanisms

Three molecular mechanisms may affect both interfaces. *Glycocalyx degradation*: TNF-*α*-activated heparanase drives syndecan-1 shedding; pulmonary consequences include neutrophil adhesion, alveolarcapillary permeability amplification, and ARDS; systemic consequences include capillary leak and functional short-circuit opening (Uchimido et al., 2019). Elevated plasma syndecan-1 is associated with ARDS, circulatory failure, and mortality (Colbert and Schmidt, 2016; Zinter et al., 2018). *Heterogeneous iNOS overexpression*: spatially heterogeneous iNOS activation may contribute to V/Q maldistribution pulmonarily and microcirculatory perfusion maldistribution systemically (Gierhardt et al., 2021; Rovas et al., 2019). *Neutrophil extracellular traps (NETs)*: pulmonary NETs promote microthrombus formation and alveolar-capillary barrier disruption; systemic NETs cause microvascular occlusion via the plateletthrombin axis (Lefranc¸ais et al., 2018). PAD4 deficiency or DNase treatment reduces NET-mediated vascular injury in experimental models, supporting rather than proving a mechanism spanning both interfaces (McDonald et al., 2017).

These observations suggest that ARDS and distributive shock may share upstream pathways. No randomised trial targeting glycocalyx integrity, selective iNOS inhibition, or NETs degradation has adopted dual-interface ICV as a combined primary endpoint.

The proposed equivalence has important limitations. “Systemic functional shunt” is not widely accepted terminology, and low oxygen extraction, elevated venous saturation, or hyperlactataemia can also reflect low metabolic demand, mitochondrial dysfunction, adrenergic glycolysis, or impaired clearance. Moreover, IDF/SDF and NIRS sample regional tissue, and no validated bedside measure quantifies a systemic ICV fraction. The framework should therefore be regarded as a testable hypothesis rather than a diagnostic or therapeutic target.

## 5 Conclusions and perspectives

The ICV framework rests on two distinctions. First, cardiac output and effective gas exchange capacity are not interchangeable: pulmonary shunt or microcirculatory maldistribution may persist while total flow is preserved. Second, anatomical and functional abnormalities differ in their susceptibility to intervention: supportive measures may improve the latter’s physiological consequences without correcting their upstream cause.

Validation should proceed in stages. A prospective methods study should first establish reproducibility and confounding of candidate systemic measures such as PVD, HI, NIRS-VOT, O_2_ER, CRT, and CO_2_-derived indices. A subsequent multicentre cohort should obtain time-synchronised pulmonary shunt and systemic microcirculatory measurements to test convergent validity, temporal responsiveness, and association with organ dysfunction. Only after a reproducible construct is defined should an interventional trial evaluate dual-interface measures, initially as mechanistic secondary endpoints.

The ICV framework’s principal utility is terminological—a single label for blood failing to participate in gas exchange at either interface. Clinical translation requires quantification: pulmonary ICV fraction has an established surrogate in *Q_s_/Q_t_*; systemic ICV fraction lacks an equivalent bedside measure. Developing such an index is the unresolved prerequisite for transitioning ICV from a conceptual construct to a prospectively testable endpoint.

## Glossary

ARISE: Australasian Resuscitation in Sepsis Evaluation
ARDS: acute respiratory distress syndrome
ATP: adenosine triphosphate
C(a-v)O_2_: arteriovenous oxygen content difference
CRT: capillary refill time
DECT: dual-energy computed tomography
EIT: electrical impedance tomography
EGDT: early goal-directed therapy
ET-1: endothelin-1
FiO_2_: fraction of inspired oxygen
HI: heterogeneity index
HPV: hypoxic pulmonary vasoconstriction
ICV: ineffective circulating volume
IDF: incident dark-field
iNO: inhaled nitric oxide
iNOS: inducible nitric oxide synthase
K_v_: voltage-gated potassium channel
MAP: mean arterial pressure
NETs: neutrophil extracellular traps
NIRS: near-infrared spectroscopy
NIRS-VOT: near-infrared spectroscopy with vascular occlusion test
NO: nitric oxide
O_2_ER: oxygen extraction ratio
P(A-a)O_2_: alveolar-arterial oxygen gradient
PAD4: peptidylarginine deiminase 4
PaO_2_: arterial partial pressure of oxygen
PAVM: pulmonary arteriovenous malformation
PEEP: positive endexpiratory pressure
PFO: patent foramen ovale
PGI_2_: prostacyclin
PPV: proportion of perfused vessels
ProCESS: Protocolized Care for Early Septic Shock
ProMISe: Protocolised Management in Sepsis
P_v-a_CO_2_: venoarterial CO_2_ difference
PVD: perfused vessel density
RBC: red blood cell
ScvO_2_: central venous oxygen saturation
SDF: sidestream dark-field
SSC: Surviving Sepsis Campaign
StO_2_: tissue oxygen saturation
SVR: systemic vascular resistance
SvO_2_: mixed venous oxygen saturation
TPBT: transpulmonary bubble transit
V/Q: ventilation-perfusion ratio
VOT: vascular occlusion test
VV-ECMO: venovenous extracorporeal membrane oxygenation.

## References

[B1] Albert R. K. Jobe A. (2012). Gas exchange in the respiratory distress syndromes. Compr. Physiol. 2, 1585–1617. doi: 10.1002/j.2040-4603.2012.tb00436.x 23723019

[B2] Aykut G. Veenstra G. Scorcella C. Ince C. Boerma C. (2015). Cytocam-idf (incident dark field illumination) imaging for bedside monitoring of the microcirculation. Intensive Care Med. Exp. 3, 40. doi: 10.1186/s40635-015-0040-7 26215807 PMC4512989

[B3] Bateman R. M. Sharpe M. D. Ellis C. G. (2003). Bench-to-bedside review: microvascular dysfunction in sepsis, hemodynamics, oxygen transport, and nitric oxide. Crit. Care 7, 359–373. 10.1186/cc2353 PMC270719 12974969

[B4] Boerma E. C. Koopmans M. Konijn A. Kaiferova K. Bakker A. J. van Roon E. N. . (2010). Effects of nitroglycerin on sublingual microcirculatory blood flow in patients with severe sepsis/septic shock after a strict resuscitation protocol: a double-blind randomized placebo controlled trial. Crit. Care Med. 38, 93–100. doi: 10.1097/ccm.0b013e3181b02fc1 19730258

[B5] Boissier F. Razazi K. Thille A. W. Roche-Campo F. Leon R. Vivier E. . (2015). Echocardiographic detection of transpulmonary bubble transit during acute respiratory distress syndrome. Ann. Intensive Care 5, 5. doi: 10.1186/s13613-015-0046-z 25859416 PMC4388070

[B6] Busana M. Rau A. Lazzari S. Gattarello S. Cressoni M. Biggemann L. . (2024). Causes of hypoxemia in COVID-19 acute respiratory distress syndrome: a combined multiple inert gas elimination technique and dual-energy computed tomography study. Anesthesiology 140, 251–260. doi: 10.1097/aln.0000000000004757 37656772

[B7] Caplan M. Goutay J. Bignon A. Jaillette E. Favory R. Mathieu D. . (2021). Almitrine infusion in severe acute respiratory syndrome coronavirus 2-induced acute respiratory distress syndrome: a single-center observational study. Crit. Care Med. 49, e191–e198. doi: 10.1016/0140-6736(93)93067-b 33093279

[B8] Colbert J. F. Schmidt E. P. (2016). Endothelial and microcirculatory function and dysfunction in sepsis. Clinics Chest Med. 37, 263–275. doi: 10.1016/j.ccm.2016.01.009 27229643 PMC4884305

[B9] Cornet A. D. Hofstra J. J. Swart E. L. Girbes A. R. Juffermans N. P. (2010). Sildenafil attenuates pulmonary arterial pressure but does not improve oxygenation during ards. Intensive Care Med. 36, 758–764. doi: 10.1007/s00134-010-1754-3 20130830 PMC2850529

[B10] De Backer D. Creteur J. Dubois M. J. Sakr Y. Koch M. Verdant C. . (2006). The effects of dobutamine on microcirculatory alterations in patients with septic shock are independent of its systemic effects. Crit. Care Med. 34, 403–408. doi: 10.1097/01.ccm.0000198107.61493.5a 16424721

[B11] Donati A. Damiani E. Domizi R. Scorcella C. Carsetti A. Tondi S. . (2016). Near-infrared spectroscopy for assessing tissue oxygenation and microvascular reactivity in critically ill patients: a prospective observational study. Crit. Care 20, 311. doi: 10.1186/s13054-016-1500-5 27716370 PMC5045573

[B12] Elbers P. W. Ince C. (2006). Mechanisms of critical illness: classifying microcirculatory flow abnormalities in distributive shock. Crit. Care 10, 221. 10.1186/cc4969 PMC1750971 16879732

[B13] Evans L. Rhodes A. Alhazzani W. Antonelli M. Coopersmith C. M. French C. . (2021). Surviving sepsis campaign: international guidelines for management of sepsis and septic shock 2021. Intensive Care Med. 47, 1181–1247. doi: 10.1007/s00134-021-06506-y 34599691 PMC8486643

[B14] Feiner J. R. Weiskopf R. B. (2017). Evaluating pulmonary function: an assessment of pao2/fio2. Crit. Care Med. 45, e40–e48. doi: 10.1097/ccm.0000000000002017 27618274

[B15] Frerichs I. Amato M. B. P. van Kaam A. H. Tingay D. G. Zhao Z. Grychtol B. . (2017). Chest electrical impedance tomography examination, data analysis, terminology, clinical use and recommendations: consensus statement of the TRanslational EIT developmeNt stuDy group. Thorax 72, 83–93. doi: 10.1136/thoraxjnl-2016-208357 27596161 PMC5329047

[B16] Gierhardt M. Pak O. Walmrath D. Seeger W. Grimminger F. Ghofrani H. A. . (2021). Impairment of hypoxic pulmonary vasoconstriction in acute respiratory distress syndrome. Eur. Respir. Rev. 30, 210059. doi: 10.1183/16000617.0059-2021 34526314 PMC9489056

[B17] Hernandez G. Ospina-Tascon G. A. Damiani L. P. Estenssoro E. Dubin A. Hurtado J. . (2019a). Effect of a resuscitation strategy targeting peripheral perfusion status vs serum lactate levels on 28-day mortality among patients with septic shock: the ANDROMEDA-SHOCK randomized clinical trial. JAMA 321, 654–664. 10.1001/jama.2019.0071 PMC6439620 30772908

[B18] Hernandez G. Teboul J. L. Bakker J. (2019b). Norepinephrine in septic shock. Intensive Care Med. 45, 687–689. doi: 10.1097/01.ccm.0001102816.46938.cb 30631902

[B19] Hjortrup P. B. Haase N. Bundgaard H. Thomsen S. L. Winding R. Pettilä V. . (2016). Restricting volumes of resuscitation fluid in adults with septic shock after initial management: the CLASSIC randomised, parallel-group, multicentre feasibility trial. Intensive Care Med. 42, 1695–1705. doi: 10.1007/s00134-016-4500-7 27686349

[B20] Homma S. Messé S. R. Rundek T. Sun Y. P. Franke J. Davidson K. . (2016). Patent foramen ovale. Nat. Rev. Dis. Primers 2, 15086. doi: 10.1002/9781444306262.ch16 27188965

[B21] Ince C. (2005). The microcirculation is the motor of sepsis. Crit. Care 9, S13–S19. 10.1186/cc3753 PMC3226164 16168069

[B22] Ince C. (2015). Hemodynamic coherence and the rationale for monitoring the microcirculation. Crit. Care 19, S8. doi: 10.1186/cc14726 26729241 PMC4699073

[B23] Ince C. Boerma E. C. Cecconi M. De Backer D. Shapiro N. I. Duranteau J. . (2018). Second consensus on the assessment of sublingual microcirculation in critically ill patients: results from a task force of the European Society of Intensive Care Medicine. Intensive Care Med. 44, 281–299. doi: 10.1007/s00134-018-5070-7 29411044

[B24] Ince C. Sinaasappel M. (1999). Microcirculatory oxygenation and shunting in sepsis and shock. Crit. Care Med. 27, 1369–1377. doi: 10.1097/00003246-199907000-00031 10446833

[B25] Joffre J. Hellman J. Ince C. Ait-Oufella H. (2020). Endothelial responses in sepsis. Am. J. Respir. Crit. Care Med. 202, 361–370. doi: 10.1164/rccm.201910-1911tr 32101446

[B26] Kaisers U. Busch T. Deja M. Donaubauer B. Falke K. J. (2003). Selective pulmonary vasodilation in acute respiratory distress syndrome. Crit. Care Med. 31, S337–S342. doi: 10.1097/01.ccm.0000057913.45273.1a 12682462

[B27] Kanoore Edul V. S. Ince C. Vazquez A. R. Rubatto P. N. Espinoza E. D. Welsh S. . (2016). Similar microcirculatory alterations in patients with normodynamic and hyperdynamic septic shock. Ann. Am. Thorac. Soc. 13, 240–247. doi: 10.1513/annalsats.201509-606oc 26624559

[B28] Karbing D. S. Panigada M. Bottino N. Spinelli E. Protti A. Rees S. E. . (2020). Changes in shunt, ventilation/perfusion mismatch, and lung aeration with peep in patients with ards: a prospective single-arm interventional study. Crit. Care 24, 111. doi: 10.1186/s13054-020-2834-6 32293506 PMC7092565

[B29] Leali M. Marongiu I. Spinelli E. Chiavieri V. Perez J. Panigada M. . (2024). Absolute values of regional ventilation-perfusion mismatch in patients with ards monitored by electrical impedance tomography and the role of dead space and shunt compensation. Crit. Care 28, 241. doi: 10.1186/s13054-024-05033-8 39010228 PMC11251389

[B30] Lefranc¸ais E. Mallavia B. Zhuo H. Calfee C. S. Looney M. R. (2018). Maladaptive role of neutrophil extracellular traps in pathogen-induced lung injury. JCI Insight 3, e98178. 10.1172/jci.insight.98178 PMC5821185 29415887

[B31] Lima A. van Bommel J. Jansen T. C. Ince C. Bakker J. (2009). Low tissue oxygen saturation at the end of early goal-directed therapy is associated with worse outcome in critically ill patients. Crit. Care 13, S13. doi: 10.1186/cc8011 19951385 PMC2786115

[B32] McDonald B. Davis R. P. Kim S. J. Tse M. Esmon C. T. Kolaczkowska E. . (2017). Platelets and neutrophil extracellular traps collaborate to promote intravascular coagulation during sepsis in mice. Blood 129, 1357–1367. doi: 10.1182/blood-2016-09-741298 28073784 PMC5345735

[B33] Mesquida J. Saludes P. Gruartmoner G. Espinal C. Torrents E. Baigorri F. . (2015). Central venous-to-arterial carbon dioxide difference combined with arterial-to-venous oxygen content difference is associated with lactate evolution in the hemodynamic resuscitation process in early septic shock. Crit. Care 19, 126. doi: 10.1186/s13054-015-0858-0 25888382 PMC4384363

[B34] Mouncey P. R. Osborn T. M. Power G. S. Harrison D. A. Sadique M. Z. Grieve R. D. . (2015). Trial of early, goal-directed resuscitation for septic shock. N. Engl. J. Med. 372, 1301–1311. doi: 10.1056/nejmoa1500896 25776532

[B35] Peake S. L. Delaney A. Bailey M. Bellomo R. Cameron P. A. Cooper D. J. . (2014). Goal-directed resuscitation for patients with early septic shock. N. Engl. J. Med. 371, 1496–1506. 10.1056/NEJMoa1404380 25272316

[B36] Prescott H. C. Antonelli M. Alhazzani W. Møller M. H. Alshamsi F. Azevedo L. C. P. . (2026). Surviving sepsis campaign: international guidelines for management of sepsis and septic shock 2026. Intensive Care Med. 52, 863–936. doi: 10.1007/s00134-026-08361-1 41870560

[B37] Pries A. R. Höpfner M. le Noble F. Dewhirst M. W. Secomb T. W. (2010). The shunt problem: control of functional shunting in normal and tumour vasculature. Nat. Rev. Cancer 10, 587–593. doi: 10.1038/nrc2895 20631803 PMC3109666

[B38] Quintel M. Bartlett R. H. Grocott M. P. W. Combes A. Ranieri M. V. Baiocchi M. . (2020). Extracorporeal membrane oxygenation for respiratory failure. Anesthesiology 132, 1257–1276. doi: 10.1097/aln.0000000000003221 32149776

[B39] Radermacher P. Maggiore S. M. Mercat A. (2017). Gas exchange in acute respiratory distress syndrome. Am. J. Respir. Crit. Care Med. 196, 964–984. doi: 10.1007/978-3-642-83010-5_21 28406724

[B40] Rovas A. Seidel L. M. Vink H. Pohlkötter T. Pavenstädt H. Ertmer C. . (2019). Association of sublingual microcirculation parameters and endothelial glycocalyx dimensions with illness severity and outcome in icu patients: a prospective observational study. Crit. Care 23, 260. doi: 10.1186/s13054-019-2542-2 31340868 PMC6657098

[B41] Sanfilippo F. Santonocito C. Morelli A. Foex P. (2022). A systematic review and meta-analysis of the association of sublingual microcirculation and poor outcome in septic patients. Intensive Care Med. 48, 1049–1063.

[B42] Segal S. S. (2005). Regulation of blood flow in the microcirculation. Microcirculation 12, 33–45. doi: 10.1080/10739680590895028 15804972

[B43] Spronk P. E. Ince C. Gardien M. J. Mathura K. R. Oudemans-van Straaten H. M. Zandstra D. F. (2002). Nitroglycerin in septic shock after intravascular volume resuscitation. Lancet 360, 1395–1396. doi: 10.1016/s0140-6736(02)11393-6 12423989

[B44] Uchimido R. Schmidt E. P. Shapiro N. I. (2019). The glycocalyx: a novel diagnostic and therapeutic target in sepsis. Crit. Care 23, 16. doi: 10.1186/s13054-018-2292-6 30654825 PMC6337861

[B45] Vallet B. Pinsky M. R. Cecconi M. (2013). Resuscitation of patients with septic shock: please “mind the gap”! Intensive Care Med. 39, 1653–1655. doi: 10.1007/s00134-013-2998-5 23812340 PMC3732761

[B46] Velthuis S. Buscarini E. Gossage J. R. Snijder R. J. Mager J. J. Post M. C. (2015). Clinical implications of pulmonary shunting on saline contrast echocardiography. J. Am. Soc. Echocardiography 28, 255–263. doi: 10.1016/j.echo.2014.12.008 25623000

[B47] Vincent J. L. De Backer D. (2013). Circulatory shock. N. Engl. J. Med. 369, 1726–1734. doi: 10.1056/nejmra1208943 24171518

[B48] Wang R. Wang W. Tang X. Qi Z. Li T. Liu Y. . (2025). Association between ventilationperfusion matching improvement during initial prone positioning and icu mortality in patients with moderate to severe ards: a prospective two-center study. Ann. Intensive Care 15, 69. doi: 10.1186/s13613-025-01489-1 40394400 PMC12092903

[B49] Wang Y. X. Zhong M. Dong M. H. Song J. Q. Zheng Y. J. Wu W. . (2022). Prone positioning improves ventilation-perfusion matching assessed by electrical impedance tomography in patients with ards: a prospective physiological study. Crit. Care 26, 154. doi: 10.21203/rs.3.rs-1388270/v1 35624489 PMC9137443

[B50] Wu Y. Wang A. Peng C. Zou X. Zhang H. Gao X. . (2025). Predicting early prone position ventilation responsiveness in patients with acute respiratory distress syndrome based on electrical impedance tomography: a prospective study. Crit. Care 30, 21. doi: 10.1186/s13054-025-05778-w 41345696 PMC12797626

[B51] Yealy D. M. Kellum J. A. Huang D. T. Barnato A. E. Weissfeld L. A. Pike F. ProCESS Investigators . (2014). A randomized trial of protocol-based care for early septic shock. N. Engl. J. Med. 370, 1683–1693. 10.1056/NEJMoa1401602 PMC4101700 24635773

[B52] Yuan X. Zhao Z. Chao Y. Chen D. Chen H. Zhang R. . (2023). Effects of early versus delayed application of prone position on ventilation-perfusion mismatch in patients with acute respiratory distress syndrome: a prospective observational study. Crit. Care 27, 462. doi: 10.1186/s13054-023-04749-3 38012731 PMC10683149

[B53] Zinter M. S. Bhatt S. R. Spicer A. C. Matthay M. A. Sapru A. (2018). Plasma syndecan-1 protein concentrations in pediatric acute respiratory distress syndrome: effects of the primary diagnosis and association with secondary outcomes. Crit. Care Med. 46, e653–e660.

